# A review of important heavy metals toxicity with special emphasis on nephrotoxicity and its management in cattle

**DOI:** 10.3389/fvets.2023.1149720

**Published:** 2023-03-29

**Authors:** Ifrah Tahir, Khalid Ali Alkheraije

**Affiliations:** ^1^Department of Parasitology, University of Agriculture, Faisalabad, Pakistan; ^2^Department of Veterinary Medicine, College of Agriculture and Veterinary Medicine, Qassim University, Buraidah, Saudi Arabia

**Keywords:** lead, cattle, arsenic, cadmium, acute kidney failure, oxidative stress, geographic distribution

## Abstract

Toxicity with heavy metals has proven to be a significant hazard with several health problems linked to it. Heavy metals bioaccumulate in living organisms, pollute the food chain, and possibly threaten the health of animals. Many industries, fertilizers, traffic, automobile, paint, groundwater, and animal feed are sources of contamination of heavy metals. Few metals, such as aluminum (Al), may be eliminated by the elimination processes, but other metals like lead (Pb), arsenic (As), and cadmium (Ca) accumulate in the body and food chain, leading to chronic toxicity in animals. Even if these metals have no biological purpose, their toxic effects are still present in some form that is damaging to the animal body and its appropriate functioning. Cadmium (Cd) and Pb have negative impacts on a number of physiological and biochemical processes when exposed to sub-lethal doses. The nephrotoxic effects of Pb, As, and Cd are well known, and high amounts of naturally occurring environmental metals as well as occupational populations with high exposures have an adverse relationship between kidney damage and toxic metal exposure. Metal toxicity is determined by the absorbed dosage, the route of exposure, and the duration of exposure, whether acute or chronic. This can lead to numerous disorders and can also result in excessive damage due to oxidative stress generated by free radical production. Heavy metals concentration can be decreased through various procedures including bioremediation, pyrolysis, phytoremediation, rhizofiltration, biochar, and thermal process. This review discusses few heavy metals, their toxicity mechanisms, and their health impacts on cattle with special emphasis on the kidneys.

## Introduction

**Figure 1:**
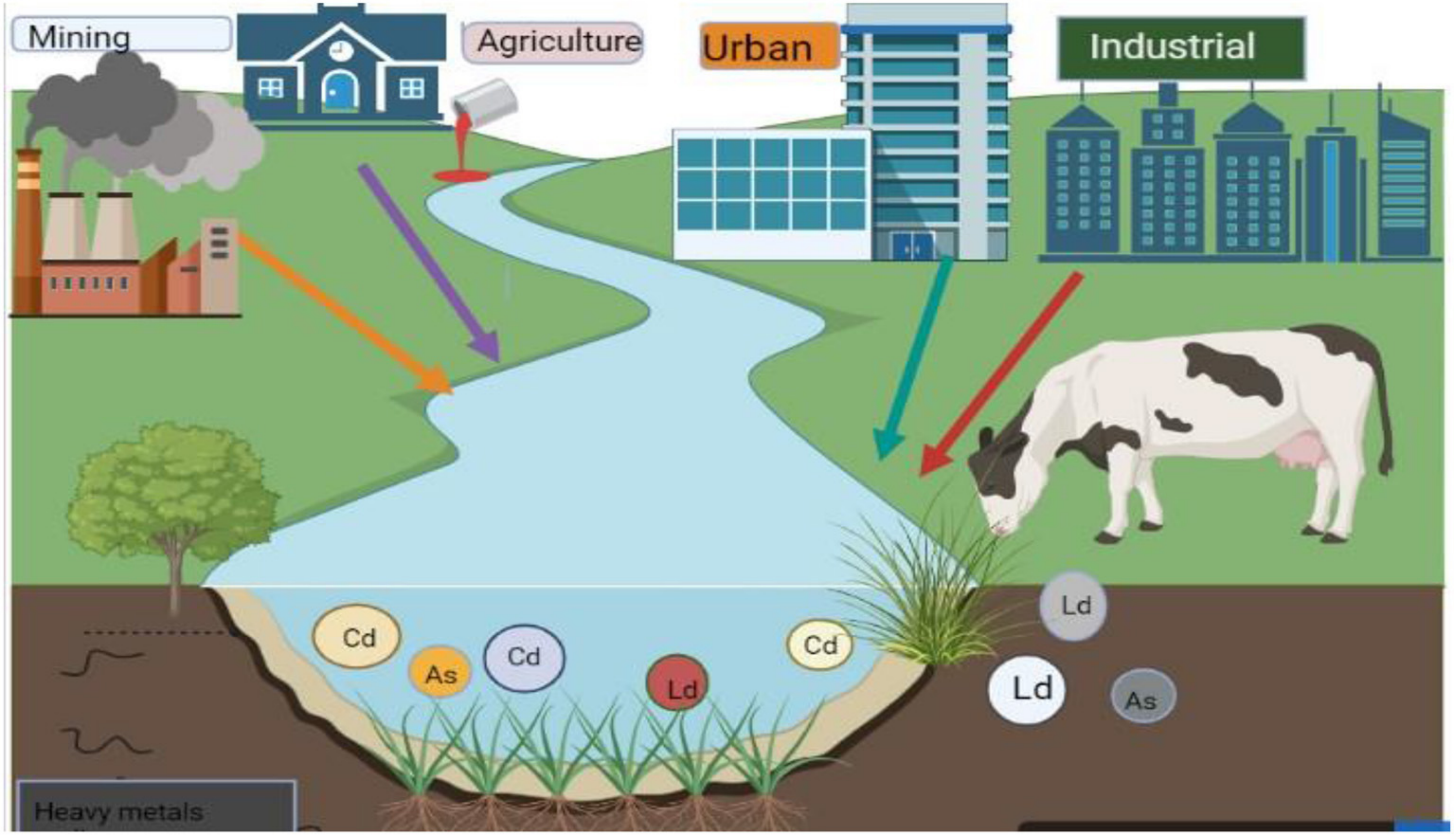
Source of heavy metals toxicity in animals (derived from bio render).

The existence of heavy metals in animal feed and water is injurious to animal health because of their bioaccumulation (1, 2). Few heavy metals including As, Cd, and Pb are well known for their toxicity, while others such as zinc (Zn), copper (Cu), cobalt (Co), manganese (Mn), iron (Fe), magnesium (Mg), and selenium (Se) are necessary for key physiological functions in trace amounts (3–6). Among all metals, Pb, As, and Cd have more negative effects on both animal and human health (7, 8). Mercury (Hg), Cd, and Pb are examples of toxic heavy metals that are dangerous even at very low doses and have no known biological benefits (9). Pb and Cd negatively impact several biochemical and physiological processes when exposed to sub-lethal doses (10, 11). Ruminants are often exposed to environmental poisons that are toxic at certain dosages in a number of areas (12, 13). However, they are particularly vulnerable to Cd, Pb, As, and Flouride (F^−^) environmental poisoning (14, 15). Domestic animals live in the same environment as people and are susceptible to heavy metals mostly through the plants, feed, soil, and water in their environment (16, 17). To a lesser extent, they are exposed through the air they breathe because of industrial and traffic pollution (18). The secondary cause of heavy metal contamination in animals is the use of pesticides, insecticides, and fertilizers in agricultural fields (19–21). Due to their many industrial, technological, domestic, medicinal, and agricultural applications, the risk of heavy metals exposure has significantly increased in the modern era as shown in Figure 1 (22–24). Animal feeds which might be one of the main sources of these heavy metal contaminations in animals have been found to contain higher amounts of heavy metals like Pb and As (25–27).

Contaminants of heavy metals enter the food chain through agriculture and industry (28, 29). These substances have a significant potential for acute toxicity. Because they are hazardous metals, land toxic metals can enter plants and accumulate within them (30, 31). The susceptibility of animals and livestock to toxic metals is affected by many factors (32, 33), of which the mixture of necessary and harmful components is possibly one of the most significant (34–36). Toxicity varies according to the animal's trace element metabolic state, and toxic metals also have an impact on the metabolism of trace elements.

Heavy metals are toxicants for edible offal and meat (37, 38). They pose a risk to animal health since they can result in conditions that affect kidney function as well as the cardiovascular and nervous systems and damage different organs such as the reproductive system, nervous system, the respiratory system, the liver, the gastrointestinal tract, and the endocrine system (39, 40). The toxicants Pb, As, and Cd are common and have been linked to kidney damage at high exposure levels (41, 42). The nephrotoxic effects of Pb, As, and Cd are well established and have high amounts of naturally occurring environmental metals; in addition, occupational populations with a high level of exposure have an adverse relationship between kidney damage and toxic metal exposure (43, 44). Heavy metals have mutagenicity, teratogenicity, and carcinogenicity; they induce poor body conditions, reduced reproduction rate, and lead to immunosuppression in domestic animals even at lower dosages (45, 46) because heavy metals easily cross food chains and are not recognized to perform any vital biological functions (47, 48). Toxic elements like Cd, Pb, Hg, and As can contaminate milk (49, 50). Livestock production may be negatively impacted by exposure to either excessive levels of harmful metals like Pb and Cd or inadequate amounts of vital trace elements like molybdenum and selenium (51–54). Livestock is valued highly in different regions of the world (55, 56). Approximately 1.3 billion habitats worldwide live in developing countries where their source of income indirectly or directly depends on livestock (57–60).

Metals in their ionic form can interact with biological systems and toxicological targets in a wide range of ways, which chemically speaking can make them very reactive (24, 61–63). The main livestock species affected by metals poisoning in this context is cattle, which are mostly fed locally grown fodder (64, 65). To assess the potential impacts of pollutants on livestock themselves and to quantify contaminant consumption in people, it is crucial to be aware of the levels of hazardous metals in cattle (66–69). After the energy sector, agricultural production (mostly the manufacturing of ruminant milk and meat) is responsible for the greatest greenhouse gas emissions, which have a negative influence on the environment (70, 71). Because of the changing environment, there is a constant requirement for the supply of nutritious feed for animals, especially cattle (72–74). This review aimed to comprehensively present heavy metals toxicity mechanism and effects, with a special emphasis on the disorders of the kidney system and the prevention of heavy metal contamination in cattle exposed to heavy metals.

## Heavy metals: Their toxicity mechanism and effects

Metals are entering the environment at an increasing rate due to industrialization. These metals are permanent because the environment cannot decay them. They eventually make their way into cattle tissue through the meal, where they first enter (51, 75).

### Lead (Pb)

Lead is a chemical belonging to the carbon group of the periodic table with the symbol Pb and the Latin name Plumbum, which means “the liquid silver.” Pb has an atomic number of 82, and it was the first chemical with a specific type of toxicity. As one of the most dangerous and ubiquitous environmental contaminants, Pb affects all biological systems when it comes into contact with food, drink, and air (76). Exposure to Pb causes clinical pathological changes by raising toxicity in the endocrine system and the kidney (77).

**Figure 2:**
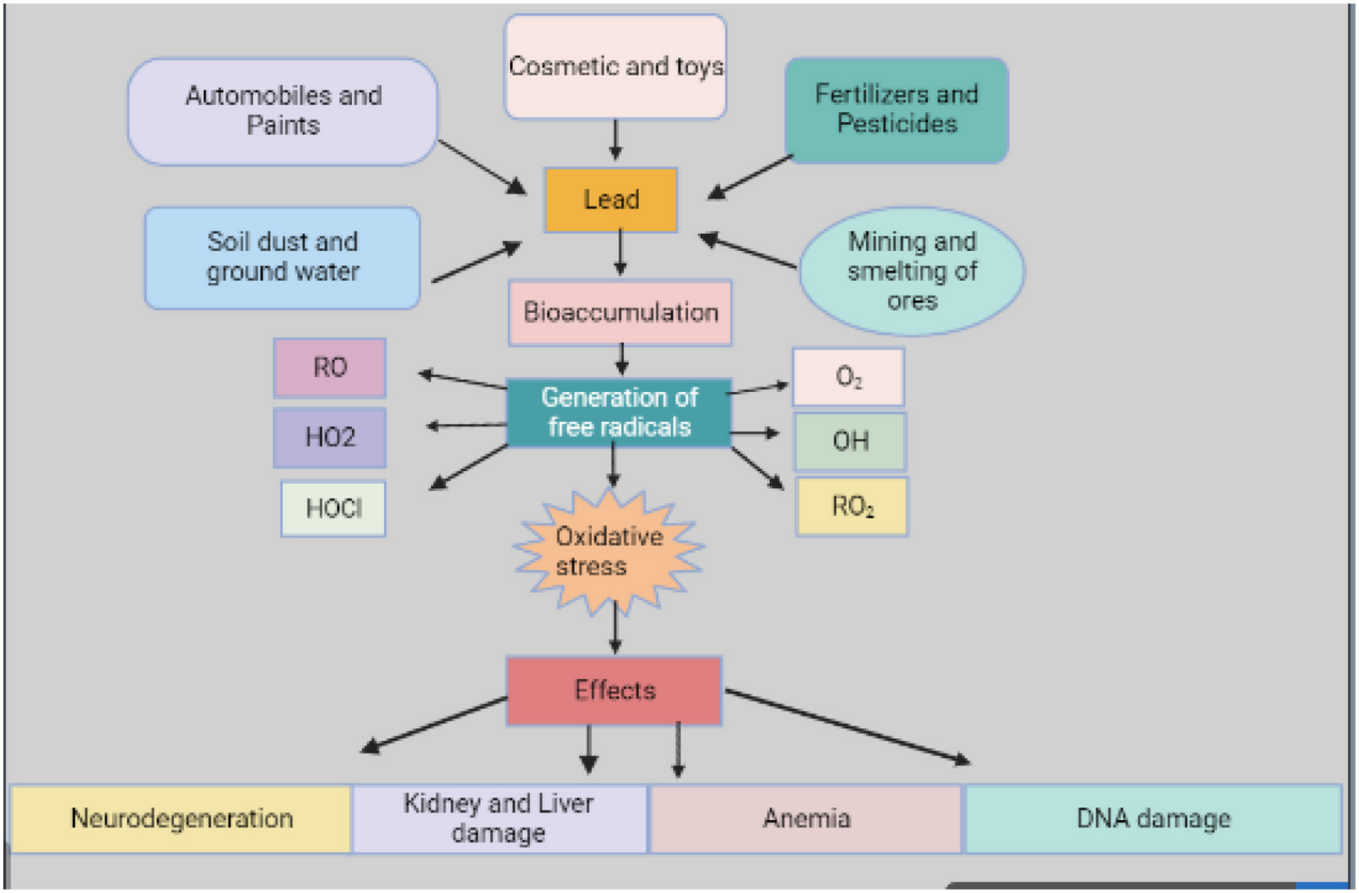
Source and effects of lead (derived from bio render).

Pb is considered one of the major environmental toxins in industrial areas of the world and animals are frequently exposed to it (78, 79). Numerous environmental factors including industrial pollution, agricultural practices (80), cosmetics, automobiles, paints, and contaminated feed and soil (Figure 2) can cause Pb poisoning, which is especially common in animals (81, 82). Accumulated Pb is toxic in its chemical composition whether it is ingested or consumed in feed or water (83).

Orally administered Pb is only minimally absorbed by the host. However, continuous exposure to Pb, even at low levels, and due to its slower rate of removal, dangerous levels of Pb can accumulate in tissues (84), which is due to an increase in reactive oxygen species (ROS) such as superoxide radicals, hydroxyl radicals, lipid peroxides, and hydrogen peroxide (85). In mammals, ROS is produced either by nicotinamide adenine dinucleotide phosphate oxidase or the mitochondrial electron transport chain which plays a role in controlling genomic stability, cell proliferation, and differentiation (82, 86). Increased ROS production occurs in many pathologic situations, including premature senescence and hematopoietic stem cell and oxidative stress due to Pb exposure, though induced hematopoietic stem cell function remains unclear (87, 88). There are approximately forty heavy metals that can be combined with a broad variety of organic molecules and powerful enzyme inhibitors due to their interaction with the ligand present in the protein and inactivate the system cell of enzymes (83, 89).

#### Effect of lead on cattle

Numerous clinical signs of Pb exposure in cattle have been noted in toxicological studies (90). Young calves of beef cattle find automotive and other mineral oils extremely appealing; hence, they are more likely to be harmed (91). However, the risk of acute exposure in cattle owing to grease and Pb-contaminated engine oil has decreased as Pb use is limited in many countries (92). Acute Pb toxicity in buffaloes and cattle affecting essential trace mineral profiles was caused by contamination of vegetation and pastures near battery manufacturing smelters (battery recycling units) and lead zinc smelters (61, 93, 94). Due to their innate eating habits, cattle are more likely to get poisoned. Hungry cattle eat everything and their chances of ingesting objects containing lead are very high (95). It acts similar to calcium in the body and builds up in the kidney, the liver, and other tissues (96, 97). Clinically, poisoned cattle typically exhibit indications of malnutrition, emaciation, muscle loss, aberrant fetal development, opaque hair, and moderate anemia and thickening of phalange epiphyses (78, 98, 99). Additionally, according to other studies, Pb-poisoned cattle exhibit ataxia, paresis of the hypoglossal nerve, severe depression, muscle twitching, convulsions, coma, death, and respiratory failure (83, 100). Pb is a tissue toxin that accumulates over time and is stored throughout the body but especially in the bones, the liver, the kidney, and the brain (101, 102). A primary component of ingested blood Pb burden that raises blood Pb levels is stored Pb in the body (61). An additional significant source of Pb exposure in cattle is grease from machinery and empty paint cans (83, 103, 104).

Chelation therapy for mercury and lead poisoning can occasionally be fatal because the Pb deposit can cause an abrupt influx of lead into the blood, severely damaging the kidney and the brain (83, 96). Most cases of Pb poisoning are either acute or chronic (105, 106). The death rate from Pb poisoning might reach 100% in cases of acute Pb poisoning (107, 108). The indicators of acute Pb poisoning in cattle appear suddenly, and the animal may pass away in the pasture within 24 h (109, 110).

#### Mechanism of action of lead on kidney

Renal dysfunction may result from Pb exposure at high levels (>60 g/dL) (111, 112). Even a trace amount of Pb (<10 g/dL) can cause the same issue (113, 114). Chronic and acute nephropathy are two different forms of renal dysfunction. Nuclear enclosing bodies, which comprise Pb protein complexes and degenerative alterations in the tubular epithelium, can be used to classify acute nephropathy both visually and functionally as a mechanism of decreased tubular transport (8, 11). Acute nephropathy may produce an abnormal secretion of glucose amino acids and phosphates, a combination known as Fanconi's syndrome (115), although it is not the cause of protein appearing in the urine. Chronic nephropathy, on the other hand, is easier to treat but can result in permanent morphological and functional abnormalities. It causes hyperuricemia, hypertension, and renal breakdown but is classified by glomerular and tubulointerstitial variants (116, 117).

The oxidative stress that Pb exposure induces appears to have a detrimental effect on the kidneys of cattle, leading to the development of renal toxicity (118, 119). Cattle exposed to Pb have higher levels of lipid peroxidation in their kidneys (23, 120). Long-term Pb exposure causes the kidney to produce lipid peroxidation and free radicals, which lead to a loss of membrane permeability and the inactivation of components of tubular cells (121, 122). Pb affects the amount of Glutathione (GSH) and the function of antioxidant enzymes like catalase (CAT), glucose-6-phosphate dehydrogenase (G6PD), glutathione peroxidase (GPx), superoxide dismutase (SOD), and glutathione S-transferase (GST) in cattle (123). This indicates that a considerable decrease in the antioxidant enzyme activity in renal tissues is caused by continuous oral Pb exposure (64, 124, 125). The mechanism of the effects of Pb on enzymes can be complicated given that Pb can competitively hinder bio-element absorption or bind with the SH group of proteins (64). Oxidative stress as a mechanism of Pb toxicity in the kidney shows that Pb exposure causes an increase in apoptosis in the kidney (126). The frequency of apoptotic bodies inside proximal tubular cells increased after 12 weeks of continuous lead acetate therapy (23, 127). Therefore, it is conceivable that Pb poisoning affects the gene expression of proteins involved in apoptosis. Following absorption, Pb is transferred to a variety of bodily tissues. Pb exposure causes histopathological alterations in the renal proximal tubular epithelium, which result in interstitial nephritis typically associated with hypertension (128, 129). Pb gets collected in the renal cortex's proximal involuted tubules, which exhibit morphological and biochemical signs of Pb toxicity (130). Occult Pb nephropathy may not be detected as such because acute Pb-induced kidney damage can happen without acute overdose (131). Renal function impairment occurs as a result of persistent lead buildup in the body. It was concluded that the formation of renal toxicity due to environmental lead exposure results in major pathological lesions on the kidney of cattle that appears to be influenced by oxidative stress (23, 132, 133).

### Arsenic (As)

**Figure 3:**
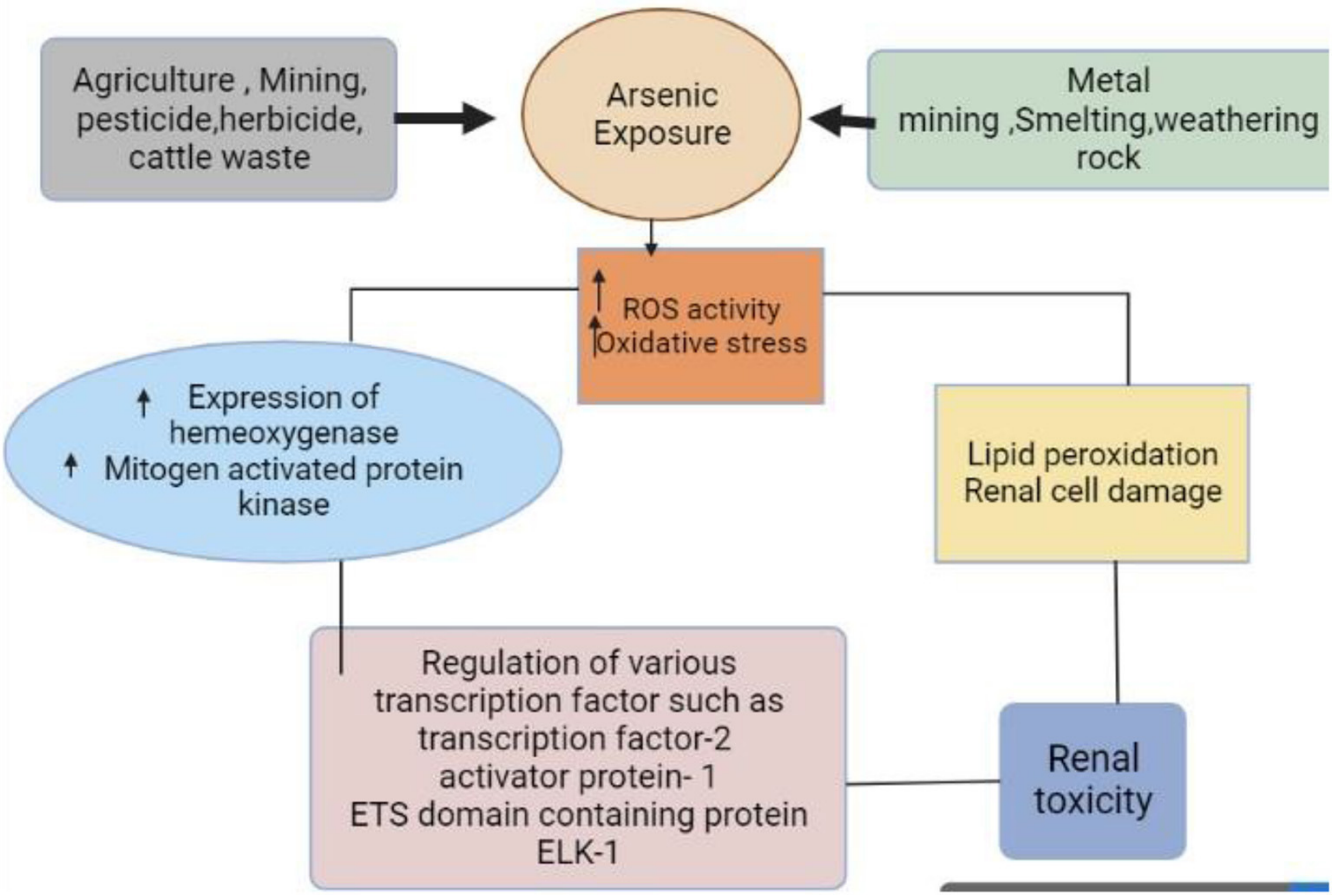
Source and mechanism of arsenic exposure (derived from bio render).

Arsenic is an environmental chemical substance of great significance to animal health (11, 134). Sodium arsenate, sodium and arsenic pentoxide, and disodium or monosodium acid are all deadly forms of As (107, 135), and their environmental contamination poses a serious health risk. Arsenic is a harmful element that is found everywhere and has become more concentrated in water and soil as shown in Figure 3 (136, 137). It can be found in inorganic, organic, pentavalent, and trivalent forms, and it can combine with a wide range of elements, including Pb, O, H, Cu, and S (31, 138). Similar to human exposure, cattle in As-affected areas are also exposed to hazardous quantities of the metal (6, 107). In places where As contamination is a problem, sources of As for animals other than drinking water include feed ingredients. Arsenic is frequently found in liquids used to dip and spray animals to control ectoparasites and cause toxicity (123). Arsenic-contaminated drinking water, feeds, vegetables, and grasses being fed to the large number of animals kept by the people severely affects the health of the animals (96). High levels of ingested As may remain in the feces, urine, blood, hair, and tissues of animals that are directly or indirectly consumed by humans. For instance, As levels of animal products are greater in polluted areas than in clean ones (17, 96). Similarly, when cattle are already As-affected, the use of their manure in agriculture and home settings causes As poisoning of the environment (139).

In terms of human health, atmospheric emissions are typically the most concerning due to the amounts involved as well as the vast dispersion and exposure risk that frequently results (140). The intake of meat and other animal products from infected cattle could expose people to the hazardous effects of As (141). However, arsenic exposure is not only due to the presence of hazardous substances but also environmental contact, which is an important element in the exposure of As (31, 142). Depending on the kinds of food that animals eat, the level of accumulation of As in varying amounts is determined. Cattle exposed to As pass on this metal in their milk and meat. For instance, in contaminated locations, As has also been found in cow meat and milk (141, 143). The WHO puts a tolerable intake of As at 3.0 g/kg body weight (144).

#### Effects of arsenic on cattle

Compared to other species, cattle are more susceptible to As poisoning (145). Cattle arsenic toxicity symptoms range from gastrointestinal to nervous system symptoms (31), severe digestive tract inflammation, weight loss, severe gastrointestinal disease, unpredictable appetite, conjunctivitis, mucosal erythematous lesions, and decreased milk production (146, 147). Kidney hyperemia and severe parasite infestation in the abomasum walls were both discovered by microscopic inspection (148, 149). The affected cattle showed decreased superoxide dismutase and catalase activities, decreased plasma nitrite and erythrocyte levels, and an increased rate of lipid peroxidation, protein carbonyl, and blood As levels in comparison to those raised in As-free areas (150, 151).

#### Mechanism of action of arsenic on kidney

Cattle exposed to As evolve tubular necrosis, glomerular sclerosis, and increased N-acetyl beta-D-glucosaminidase (NAG) concentration in urine (152–154). They also experienced DNA oxidative damage and increased oxidative stress in the kidneys (155, 156). Arsenic is believed to cause endothelial dysfunction and promote inflammation and oxidative stress (157), which may cause kidney damage; however, these are rather general mechanisms (158, 159).

The higher lipid peroxidation in the kidney after As treatment may be caused by the formation of superoxide anion radical according to the decreased SOD activity in the kidney as shown in Figure 3 (160, 161). When molecular oxygen interacts with the dimethyl arsine metabolite of dimethyl As acid, free radicals are produced. These radicals are believed to be superoxide anion radicals, which are created when dimethyl arsine reduces molecular oxygen by one electron. Arsenic induced kidney lipid peroxidation and unchanged SOD activity point to no superoxide anion buildup (162, 163).

Inorganic arsenic is methylated by two distinct enzymatic processes (164). It has been shown that trivalent inorganic As has an inhibiting influence on the second methylation process that results in the creation of dimethyl As acid (165). Although one of the detoxication steps for As is methylation, the cellular methyl group intake results in DNA hypomethylation, which alters the gene expression and causes cellular change (166). Inorganic trivalent arsenic (AS^3+^) and pentavalent arsenic (As^5+^) exhibit significantly different acute toxicity and biological processes. The renal tubules actively transport arsenate (As^5+^), and a minor portion of this form is converted to AS^3+^, the molecule that is more acutely poisonous (138, 167). It was concluded that the formation of renal toxicity by As exposure causes major kidney problems in cattle influenced by oxidative stress and lipid peroxidation (167–169).

### Cadmium (Cd)

The chemical element Cd has the atomic number 48. This silvery white and soft metal is chemically similar to the other two stable metals (zinc and mercury) in group 12 (170, 171). It is a heavy metal that is both naturally present and released as part of industrial pollution (172). Typically, it is found in minerals along with other elements like chlorine (cadmium chloride) (173–175), oxygen (cadmium oxide), or sulfur (cadmium sulfide, cadmium sulfate) (176). Although it is unknown how it operates biologically in either animals or people, it resembles the effects of other divalent metals that are crucial for a variety of biological processes (177, 178). In the aquatic environment, the presence of Cd is linked to Cd and other toxic metals being released from mining, sewage, and processing of toxic metals (179). The main sources of Cd include refined foods, water, coffee, water pipes, tea, burning coal, and chimneys (180, 181).

**Figure 4:**
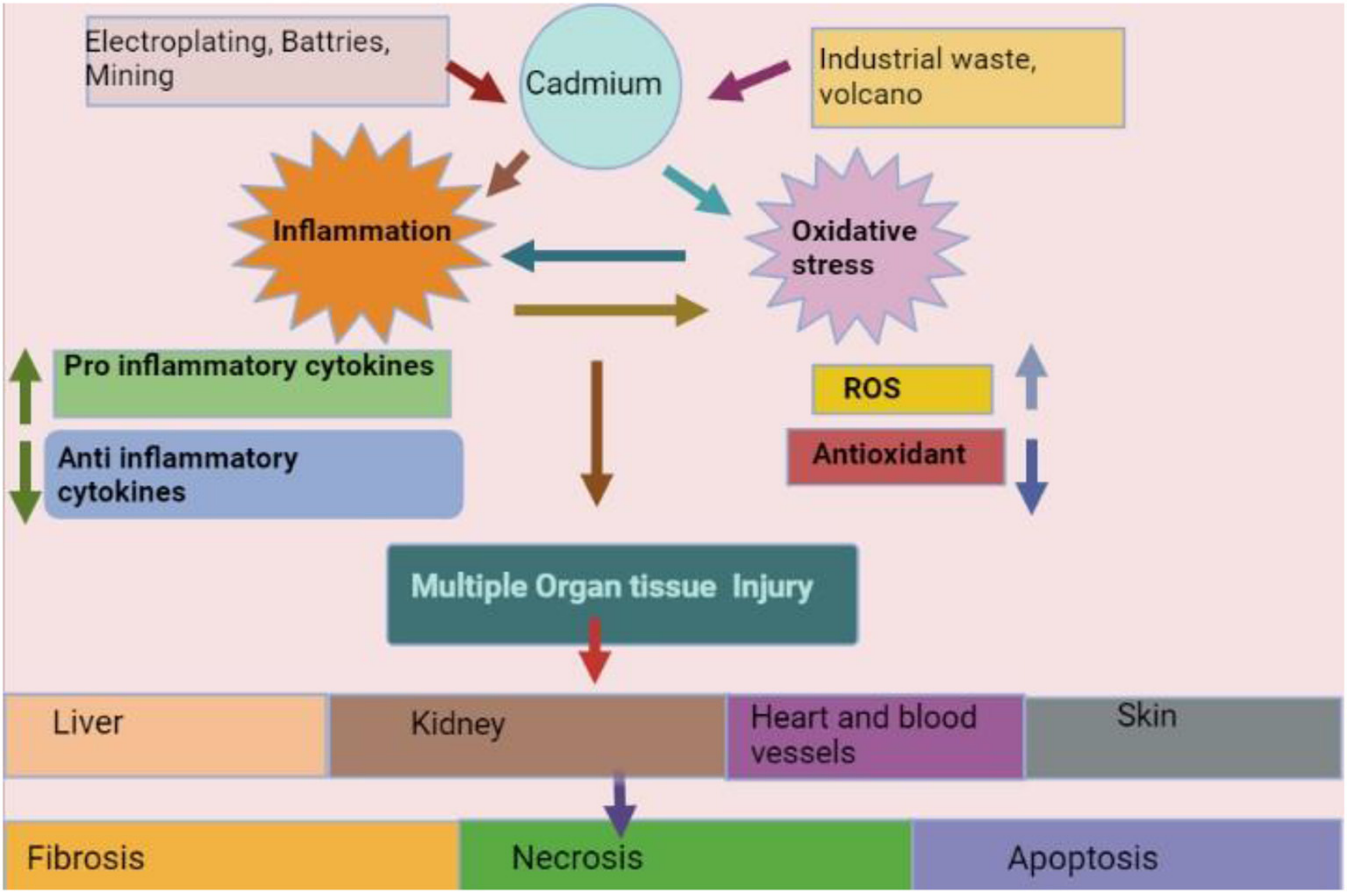
Source, mechanism, and effects of cadmium (derived from bio render).

Commercial uses for Cd include TV screens, paint pigments, lasers, batteries, cosmetics, galvanizing steel, acting as a barrier in nuclear fission, and weld sealing in lead water pipes as shown in Figure 4 (182, 183). Cd exposure occurs from taking contaminated food (e.g., organ meat, crustaceans, rice from certain areas of China and Japan, leafy vegetables) or water (Cd and Zn sealed water pipe and industrial pollution) and can cause long-term health issues. Contaminated dietary supplements and drugs are also a source of contamination (176, 184, 185). Dietary consumption of Cd varies between 40 and 50 g per day (175, 186).

#### Effects of cadmium on cattle

Almost every system in the cattle body is affected by Cd toxicity (176, 186). The toxic effects of Cd include lung damage, hypertension, hepatic injury, and kidney dysfunction (187, 188). High amounts of Cd have been discovered in the kidneys, muscles, bones, and liver of cattle in Marrakech, Morocco (186, 189) where the sewage treatment fields have disrupted the normal metabolism of trace elements and reduced the levels of Zn and Cu (171, 190). Cattle slow the cellular clearance of Cd and the ineffectiveness of cellular export systems accounts for the element's prolonged retention in storage tissues such as the colon, the liver, and the kidneys (37, 191, 192). For instance, in Nigeria, cattle grazing in regions with high Cd contamination have been shown to have high amounts of the metal in their muscles, liver, and kidneys, which also reduced the quality of their meat (31, 193).

#### Mechanisms of toxicity of cadmium on kidney

Cadmium toxicity has been observed in many organs and Cd induces tissue damage through oxidative stress (194, 195), epigenetic alterations in DNA expression (196), and upregulation and inhibition of the transport pathway (197), especially in proximal S1 region in tubules of the kidney (198, 199). The kidney is the main organ affected by Cd toxicity (171), and the S1 portion of the proximal tubule is a prime target for Cd deposition. As a result of Cd-induced oxidative damage to carrier proteins and mitochondria (181), Fanconi syndrome is characterized by clinically visible defects in protein, bicarbonate, phosphate, and amino acid reabsorption (200, 201). Approximately thirty percent of body Cd is accumulated in kidney tubule segments, with tubular injury proportional to the amount of Cd that is not bound to metallothionein (202, 203). It was concluded that Cd toxicity damages the kidney through oxidative stress.

## Geographical distribution of metal toxicity in cattle

Metal toxicity has been observed in different animals, but our focus is on cattle in this review. We found that metal toxicity is highly prevalent in cattle worldwide as detailed in Tables 1–3. Different metals have been examined in various studies among which one study measured Cd, As, and Pb concentrations in meat, kidney, and liver from 56 cattle and 438 calves slaughtered in Galicia, Spain in 1996. In cattle, the concentrations were observed as 0.057, 0.066, 0.017 mg/kg (Pb), 0.046, 0.068, 0.005 mg/kg (As), and 0.097, 0.458, 0.001 mg/kg (Cd) in meat, kidney, and liver, respectively. The concentrations of Cd, As, and Pb in cattle in Galicia infrequently exceeded the maximum acceptable limits that many nations have adopted (235).

**Table 1:** Lead toxicity observed in different organs of cattle.

**Animal**	**Organ**	**Normal range**	**Toxicity level**	**Country**	**References**
Cattle	Kidney	0.1–0.35 ppm	>0.35 ppm	Canada	(99)
Cattle	Blood	0.1 mg/kg	1.30 mg/kg	Western Canada	(78)
Cattle	Liver	0.1–1.0 mg/kg	33.5 mg/kg	Western Canada	(78)
Cattle	Kidney	0.2–1.0 mg/kg	56.3 mg/kg	Western Canada	(78)
Cattle	Kidney	30 ppm	35.7–284.5 ppm	Paraná and São Paulo, Brazil	(105)
Cattle	Body	0.48 mg/kg	2.90 mg/kg	England	(100)
Cattle	Kidney	>10 mg/kg	>30 mg/kg	England	(100)
Cattle	Liver	10 mg/kg	23.2 mg/kg	America	(204)
Cattle	Kidney	35 mg/kg	62.8 mg/kg	America	(204)
Cattle	Liver	4.99 mg/kg	47.5 mg/kg	Spain	(107)
Cattle	Kidney	5.30 mg/kg	58.3 mg/kg	Spain	(107)
Cattle	Muscle	7.12 mg/kg	50.3 mg/kg	Spain	(107)
Cattle	Blood	4.81 mg/kg	34 mg/kg	Spain	(107)
Cattle	Kidney	6.1 mg/kg	59.7 mg/kg	Deza region (NW Spain)	(205)
Cattle	Blood	0.01 μg/ml	0.60 μg/ml	India	(76)
Bovine	Kidney	0.5 mg/kg	0.8 mg/kg	China	(206)
Bovine	Kidney	0.5 mg/kg	0.1 mg/kg	China	(206)
Bovine	Muscles	0.2 mg/kg	0.001 mg/kg	China	(206)
Cattle	Kidney	0.05 μg/ml	1.04 μg/ml	Kabwe, Zambia	(207)
Cattle	Kidney	0.5 mg/kg	0.52 mg/kg	Jamaica	(37)
cattle	Kidney	0.5 mg/kg	2.64 mg/kg	Netherland	(208)
Cattle	Muscle	2.00 mg/kg	1.95 mg/kg	Croatia	(209)
Cattle	Kidney	0.172 mg/kg	0.167 mg/kg	Croatia	(209)
Cattle	Kidney	0.04–2.97 μg/g	0.92 μg/g	Canada	(210)
Cattle	Kidney	0.022–1.21 mg/kg	0.006 mg/kg	Belgium	(211)
Cattle	Kidney	0.05 ppm	0.08 ppm	India	(120)
Cattle	Kidney	0.006 mg/kg	0.002 mg/kg	Italy	(212)
Cattle	Blood	0.46 μg/g	0.54 μg/g	Pakistan	(213)

In another study, the correlation between toxic As, Cd, and Pb was examined in the kidney, muscle, blood, and liver of 494 cattle from Galicia. These interactions are most likely a result of the effects that Cd has on the synthesis of metallothionein (107, 236). In the kidney, Pb and Zn were positively correlated; however, it is unclear how they interact. Overall, the levels of Pb and As in Galician cattle do not pose a threat to animal health. However, in some areas of Galicia, up to 20% of the cattle had toxic levels of Cd in their kidney (Table 3) (235, 237).

In a study in Belgium, trace element concentrations were found in the kidney, liver, and meat of cattle that had spent more than 18 months in areas that historically had been polluted by emissions from non-ferrous metal production or in areas with high levels of metals contamination (120, 238, 239). Trace element values were calculated using coupled plasma mass spectrometry. Concentrations of Cd, As, and Pb in meat were low in cattle (240). However, cattle from the polluted areas had kidney concentrations that were, respectively, 1.8, 2.2, and 2.5 times higher than those of animals from the reference locations. The European maximum level for Cd in cattle kidneys exceeded 75% in cattle from polluted environments and 47% of kidneys from reference sites. The levels of Cd, As, and Pb in cattle livers from polluted locations were 2.3 times higher. Cattle accumulated much more Cd in the kidneys and Pb in the liver and kidneys (Table 1) (120, 186).

In another study, metal detoxification and accumulation processes were determined in cattle from unpolluted and polluted areas of Italy. Dairy cattle from farms and free-ranging cattle from nature reserves were chosen as study animals (241). The concentration of Pb, Cd, and As were determined in the kidney, muscle, blood, and liver of cattle from reference and polluted areas. Cattle from contaminated areas had higher internal concentrations of Cd, Pb, and As than cattle from reference areas (Table 2) (206, 242). In another study, the results suggested Cd as the most important metal for MT induction in the kidney. Pb and Cd were significantly higher in both cattle from polluted and breed areas (243). While Cd concentration exceeded the European level by 85% in the kidney and 40% in the liver from sampled cattle, it was higher in the kidney and the liver of cows from contaminated areas (Table 3) (222, 244).

**Table 2:** Arsenic toxicity observed in different organs of cattle.

**Animal**	**Organ**	**Normal range**	**Toxicity level**	**Country**	**References**
Cattle	Liver	4.57 mg/kg	10.02 mg/kg	Spain	(107)
Cattle	Kidney	5.03 mg/kg	15.2 mg/kg	Spain	(107)
Cattle	Muscle	3.34 mg/kg	4.25 mg/kg	Spain	(107)
Cattle	Blood	3.11 mg/kg	2.92 mg/kg	Spain	(107)
Cattle	Muscle	6.07 mg/kg	5.87 mg/kg	Croatia	(209)
Cattle	Kidney	0.033 mg/kg	0.031 mg/kg	Croatia	(209)
Cattle	Kidney	0.1 mg/kg	0.1–0.5 mg/kg	Croatia	(214)
Cattle	Kidney	0.02–0.20 μg/g	0.17 μg/g	Canada	(210)
Cattle	Liver	0.02–0.13 μg/g	0.26 μg/g	Canada	(210)
Cattle	Kidney	0.002 mg/kg	0.048 mg/kg	Netherland	(210)
Cattle	Liver	0.002 mg/kg	0.013 mg/kg	Netherland	(210)
Cattle	Meat	0.002 mg/kg	0.004 mg/kg	Netherland	(210)
Cattle	Kidney	0.002 mg/kg	0.048 mg/kg	Netherland	(215)
Cattle	Kidney	0.002 mg/kg	<0.02 mg/kg	Finland	(216)
Cattle	Kidney	0.002 mg/kg	0.034 mg/kg	Germany	(217)
Cattle	Kidney	0.002 mg/kg	0.03 mg/kg	Australia	(218)
Cattle	Kidney	0.002 mg/kg	0.018 mg/kg	Australia	(219)
Cattle	Kidney	0.001–0.147 mg/kg	0.030 mg/kg	Belgium	(211)
Cattle	Kidney	0.002 mg/kg	0.001 mg/kg	Italy	(212)

**Table 3:** Cadmium toxicity observed in different organs of the cattle.

**Animal**	**Organ**	**Normal range**	**Toxicity level**	**Country**	**References**
Cattle	Liver	6.15 mg/kg	23.4 mg/kg	Galicia, NW Spain	(107)
Cattle	Kidney	5.91 mg/kg	110 mg/kg	Spain	(107)
Cattle	Muscle	9.46 mg/kg	8.28 mg/kg	Spain	(107)
Cattle	Blood	9.17 mg/kg	1.65 mg/kg	Spain	(107)
Cattle	Kidney	0.3 mg/kg	59.7 mg/kg	Deza region (NW Spain)	(205)
Cattle	Kidney	0.01 μg/ml	0.05 μg/ml	India	(220)
Cattle	Kidney	1.0 mg/kg	2.15 mg/kg	China	(206)
Bovine	Liver	0.5 mg/kg	2.47 mg/kg	China	(206)
Bovine	Muscle	0.01 mg/kg	0.02 mg/kg	China	(206)
Bovine	Kidney	0.05 μg/ml	19.37 μg/ml	Kabwe, Zambia	(207)
Cattle	Kidney	0.1 mg/kg	9.58 mg/kg	Netherland	(208)
Cattle	Kidney	0.1 mg/kg	10.3 mg/kg	Morocco	(221)
Cattle	Kidney	0.1 mg/kg	33.1 mg/kg	Jamaica	(37)
Cattle	Liver	0.1 mg/kg	0.642 mg/kg	Belgium	(186)
Cattle	Kidney	0.1 mg/kg	4.22 mg/kg	Belgium	(186)
Cattle	Liver	0.50 mg/kg	2.655 mg/kg	Belgium	(186)
Cattle	Kidney	0.1 mg/kg	15.3 mg/kg	Belgium	(186)
Cattle	Liver	0.50 mg/kg	1.17 mg/kg	Belgium	(222)
Cattle	Kidney	0.1 mg/kg	7.99 mg/kg	Belgium	(222)
Cattle	Liver	0.50 mg/kg	0.061 mg/kg	Finland	(223)
Cattle	Kidney	0.1 mg/kg	0.35 mg/kg	Finland	(223)
Cattle	Kidney	0.1 mg/kg	0.036 mg/kg	Finland	(224)
Cattle	Kidney	0.1 mg/kg	8.63 mg/kg	Ireland	(225)
Cattle	Kidney	0.1 mg/kg	1.66 mg/kg	Netherlands	(208)
Cattle	Kidney	0.1 mg/kg	0.25 mg/kg	Poland	(226)
Cattle	Kidney	0.1 mg/kg	0.937 mg/kg	Poland	(227)
Cattle	Kidney	0.1 mg/kg	0.161 mg/kg	Spain	(228)
Cattle	Kidney	0.1 mg/kg	0.545 mg/kg	Spain	(229)
Cattle	Kidney	0.1 mg/kg	0.39 mg/kg	Sweden	(230)
Cattle	Kidney	0.1 mg/kg	0.373 mg/kg	Slovenia	(211)
Cattle	Kidney	0.1 mg/kg	0.65 mg/kg	Australia	(231)
Cattle	Kidney	0.1 mg/kg	38.3 mg/kg	China	(206)
Cattle	Kidney	0.1 mg/kg	7.92 mg/kg	Jamaica	(37)
Cattle	Kidney	0.1 mg/kg	0.1371 mg/kg	Iran	(232)
Cattle	Kidney	0.1 mg/kg	4.38 mg/kg	Morocco	(221)
Cattle	Muscle	0.348 mg/kg	0.341 mg/kg	Croatia	(209)
Cattle	Kidney	0.544 mg/kg	0.535 mg/kg	Croatia	(209)
Cattle	Kidney	2.91 μg/g	17.84 μg/g	Canada	(210)
Cattle	Kidney	0.093–4.22 mg/kg	0.002 mg/kg	Belgium	(211)
Cattle	Kidney	0.05 ppm	0.09 ppm	India	(120)
Cattle	Kidney	0.001 mg/kg	0.0008 mg/kg	Italy	(212)
Cattle	Kidney	0.41 mg/kg	11.50 mg/kg	Ethiopia	(233)
Cattle	Liver	0.06 mg/kg	0.5 mg/kg	Ethiopia	(233)
Cattle	Liver	0.46 μg/g	0.54 μg/g	Pakistan	(213)
Cattle	Kidney	0.5 mg/kg	0.34 mg/kg	Turkey	(234)

In yet another study, concentrations of Cd, As, and Pb was determined in the kidney and the liver of cattle near a lead and zinc mine in Zambia, which was ranked among the top ten contaminated places in the world. The concentration of metals was measured in the kidney and liver of 51 cattle from Kabwe and other places in Zambia (79). Maximum metal concentrations expressed in the kidney and the liver were 0.05 As, 19.37 Cd, and 1.8 Pb. Concentrations of Cd and Pb in Kabwe cattle were high than the cattle from other parts of Zambia; the mean concentration of Cd exceeded the benchmark value (Table 3) (207, 245).

Pb poisoning is commonly detected in American cattle. In one of the studies, three groups of cattle were selected from various herds that had accidentally been in contact with discarded Pb batteries in the pasture (246). Blood samples were collected from cattle and monitored for changes in Pb concentration. The herds had Pb concentrations that were indicative of acute Pb exposure (>0.35 ppm) and asymptomatic Pb toxicities; between 7% and 40% of these asymptomatic cattle were in the high normal limit (0.1–0.35 ppm) (Table 1) (99).

One study evaluated the epidemiology of acute Pb poisoning in cattle in Canada over 16 years from 1998 to 2013. Over the duration of the study, there were 525 incidents of acute Pb poisoning. The toxic level of Pb was 11.2% in 2001, reduced to 9.9% in 2006, and rose to 15.6% in 2009 (78). Cattle calves six months of age were frequently poisoned (53.5%). The mean toxic Pb concentration in the kidney, the liver, and the blood was 56.3 ± 39.7 (*n* = 61), 33.5 ± 80.5 (*n* = 172), and 1.30 ± 1.70 (*n* = 301), respectively. The mean normal Pb concentration in the kidney, the liver, and the blood was 0.41 ± 0.62 mg/kg (*n* = 64), 0.16 ± 0.63 mg/kg (*n* = 382), and 0.036 ± 0.003 mg/kg (*n* = 1,081), respectively (78).

### Toxicokinetics

The majority of industrial activities affect animals and the environment in both favorable and unfavorable ways. The energy usage mitigation measures are coupling desalination plants with renewable energy sources such as wind power, geothermal energy, tidal power and solar energy (247). Typically heavy metals removal techniques may depend on the reliability of the plants, cost, operation, concentration of competitive ions and concentration of heavy metals in water (248).

The total body kinetics that a chemical is subjected to in an organism is referred to as toxicokinetics (193, 249). A toxicant enters an organism through absorption. It is distributed throughout the organism through diffusion (250). The chemical is subsequently broken down into less dangerous metabolites, which the organism may expel or store in different regions of its body (251). A chemical toxicokinetic state can vary with prolonged exposure (252). In toxicokinetics, the type of chemical which will end up in the animal depends on the physicochemical composition of the metal and the biological makeup of the recipient organism (253, 254). Metals are absorbed into an organism either by conveyors or diffusion. Pb ion, an electrically charged particle, enters using conveyors or carriers like proteins (255). Other substances can enter intracellular compartments through damaged membranes. Another possibility is loss from the cells, which would result in a drop in intracellular concentration. The amount of toxicity felt by the organism directly depends on the uptake and reduction in intracellular concentration (256, 257).

### Biological transformation

Biological transformation is the process of transforming substances within an organism (258). Biological transformation processes show how the organism's toxicant concentrations are decreased after being ingested (259, 260). The chemical breakdown within an organism is crucial to the biological transformation process because it creates new less dangerous compounds (261). In phase 1, enzymes convert a chemical toxin through the oxidative, reductive, and hydrolytic processes (262). In phase 2, transferase enzymes involve in the transformation of chemicals formed by toxicants. At this stage, the hydrophilicity of toxicants is increased (263).

## Prevention and control of bioaccumulation of toxic metals

Physical danger could potentially injure an animal and its consumer physically; therefore, safe meat must be free of toxic metals. Soil remediation is employed to make soils more useful and therefore indirectly lower the susceptibility of animals to hazardous metals (123, 264).

### Bioremediation

Techniques for restoring soil are dependent on chemical or biological principles. Toxic metals that damage the environment are removed from water and soil through bioremediation (53, 265). This entails using microbes and plants to biologically restore the utility values of polluted areas (266, 267). As a result, the hazardous metals in plants get immobilized, preventing their proliferation. These contaminants can be absorbed by bacteria that live in harmony with these plants (268).

### Phytoremediation

Utilizing phytoremediation, landfill soils are recovered. This technique is based on the utilization of plants that take up metals from the soil or water and collect them (269, 270). The capacity of plants to store and absorb metals as well as their accessibility to the plants both affect the efficacy of phytoremediation (271, 272).

### Rhizofiltration

Rhizofiltration is a type of phytoremediation in which wastewater, surface water, and contaminated groundwater are filtered by a dense network of roots to remove toxins or surplus nutrients (273, 274). The pollutants on the root undergo both adsorption and absorption during the process (275). Rhizofiltration is used for removing heavy metals from the environment.

### Biochar

The term “biochar” refers to a material rich in carbon formed during the pyrolysis process, which is the thermochemical degradation of biomass at a temperature of roughly 700°C with little or no oxygen present (276). Biochar, which can be used in a variety of environmental applications, syngas, which is converted into electricity or heat (combined power and heat), and bio-oil, which can be used as a fuel or added to petroleum refining products, are all byproducts of pyrolysis (277–279). The best way to dispose of wastewater is biochar, which can also be utilized to enhance the soil's characteristics and fertility (280). Metals that are not eliminated during sewage treatment could be successfully decreased by adding biochar to sewage sludge (281). Pollution in wastewater is reduced by pyrolyzing it to create biochar and using it further. Biochar lowers the bioavailability of harmful metals and raises the pH of the soil (282). Additionally, biochar has the potential to enhance soil quality and drastically lower the bioavailability of hazardous metals (283). Biomass is pyrolyzed to make biochar (282).

### Pyrolysis

Pyrolysis is the thermal breakdown of organic compounds at temperatures between 300 and 900°C in an oxygen-free atmosphere (284, 285). The technique of pyrolysis involves heating sewage sludge in an inactive environment to release organic material that can subsequently be recycled (286, 287). The heavy metals are concentrated by this mechanism around carbonaceous deposits (288).

### Leachate

Any liquid that removes soluble or suspended particles and any other component of the matter it has passed through is known as leachate (289). Leachate is made up of different combinations of suspended and dissolved materials, heavy metals contaminants, inorganic and organic pollutants, and more (290, 291). To avoid the undesirable outcome of surface water and groundwater contamination, landfill leachate should be gathered and properly treated. Leachate is produced as a result of waste degradation or water access, and it can contaminate groundwater and soil (282, 283). A source of contamination in cattle is metal-containing leachate.

### Thermal process

Manure has a similar energy value to wood waste, making it a suitable source of biomass for the production of energy (292, 293). Toxic metals are stabilized during the thermal processing of biomass for energy production, which lessens their toxicity (294). Most metals remain in their low-toxicity solid phase during combustion. This procedure generates energy while preventing metal contamination (294, 295).

Several preclinical and clinical research has examined the effects of heavy metal supplementation as chelating agents to facilitate pollutants elimination or as synthetic antioxidants to mitigate the oxidative stress caused by environmental pollutants to avoid or lessen toxicity (237, 296). These therapies and approaches themselves are believed to have a variety of safety and effectiveness issues.

## Conclusion

We conclude that lead and cadmium have high toxicity in the kidney and thus lead to acute kidney disorders in cattle; however, arsenic also accumulates in the kidney but at low intensity. As regards other body parts, these heavy metals penetrate the liver and muscles, but with lower intensity compared to the kidney. Advanced technologies can reduce occupational exposure to heavy metals. Monitoring exposure and perhaps intervening to reduce subsequent exposure to heavy metals in the animals and environment can be a significant step toward prevention. There is an urgent need to decrease the concentration level of these heavy toxic metals through advanced scientific techniques such as biochar, bioremediation, and pyrolysis to minimize global economic losses. In the future, it will help develop advanced techniques to control heavy metals in cattle. Failure to reduce the exposure will lead to serious issues in the future due to the negative effects of heavy metals. National and international collaboration is essential for developing adequate heavy metal toxicity prevention strategies.

## Author contributions

IT and KAA made a substantial, direct, and intellectual contribution to the work and approved it for publication.
